# Pilot Study to Evaluate the Effect of Topical Dimethicone on Clinical Signs and Skin Barrier Function in Dogs with Naturally Occurring Atopic Dermatitis

**DOI:** 10.1155/2013/239186

**Published:** 2013-04-17

**Authors:** C. Pellicoro, R. Marsella, K. Ahrens

**Affiliations:** ^1^College of Veterinary Medicine, University of Bari, Piazza Umberto I, 1-70121 Bari, Italy; ^2^Department of Small Animal Clinical Sciences, College of Veterinary Medicine, University of Florida, 2015 SW 16th Avenue, Gainesville, FL 32610-0126, USA

## Abstract

This study investigated the effects of a skin protectant solution (dimethicone 2%) on clinical signs and skin barrier function in canine atopic dermatitis (AD). Eighteen dogs with AD were randomly divided into two groups, one received dimethicone and the other received the vehicle (cyclomethicone) on selected areas (pinnae, groin, and axillae) daily for 4 weeks. Owners and investigators were blinded regarding group allocation. Clinical efficacy was evaluated using a scoring system and skin barrier by measuring the transepidermal water loss. Twelve dogs completed the study (50% drop rate in the vehicle and 20% in the dimethicone). For clinical signs, analysis of variance showed an effect of time (*P* < 0.005; day 0 > day 28) and region (axillae < groin < pinnae) but no effect of group or group × time interaction. For transepidermal water loss, analysis of variance showed only a main effect of region (axillae > pinnae > groin). Pearson found no correlation between transepidermal water loss and clinical scores. In this pilot study dimethicone had no significant effect on clinical signs and transepidermal water loss in canine atopic dermatitis.

## 1. Introduction

Atopic diseases (asthma, allergic rhinitis, and atopic dermatitis) have been reported to be increasing commonly in humans, especially in developed countries [1]. Although there is a genetic predisposition to the development of these diseases, the rapid rise in incidence is suspected to be caused by environmental factors rather than purely by genetic factors. Environmental factors that could play an important role include the increased exposure to agents that are able to disrupt the skin barrier such as the daily use of harsh soaps and the increased exposure to dust mites, which have proteolytic enzymes that can aggravate barrier.

Numerous similarities exist between canine atopic dermatitis (CAD) and its human counterpart [2, 3], and many of the same environmental factors associated with the increasing incidence of human AD are found in the environment of dogs. Interestingly, a similar increase in the incidence of AD has been reported also in dogs [4].

In recent years, abnormal barrier function has received growing attention in the pathogenesis of AD [5, 6]. In humans with AD it has been demonstrated that skin barrier impairment is linked to both genetic mutations [7, 8] and inflammation [9]. It is hypothesized that the disturbed skin barrier allows increased penetration of environmental allergens into the skin; this promotes a T helper 2 shift that further aggravates skin barrier [10]. Much less is known in veterinary medicine about skin barrier in CAD, but there is evidence to support that cutaneous impairment also exists in atopic dogs [5, 6, 11]. Due to the importance of skin barrier dysfunction, therapeutic options aimed at skin barrier repair should be investigated.

Skin barrier function can be assessed in a noninvasive way by measuring transepidermal water loss (TEWL) [12]. TEWL is higher in human patients with AD than in normal individuals [13], both in lesional and nonlesional areas [14], and variation exists between various anatomic sites [15]. Similarly to what described in human medicine, dogs with AD have increased TEWL particularly in the sites predisposed to the development of AD [16].

Barrier cream or skin protectants act as an exogenous barrier to water loss so that more moisture is retained in the stratum corneum [17–19]. Dimethicone, also called polydimethylsiloxane, is a type of silicone oil with distinctive properties that make it a useful ingredient in many skin care products. The prefix ‘‘dimeth” refers to the two methyl groups that are attached to the silicone molecule to form dimethicone. This is one of the least complicated variants of silicone and is used most often in hair care products. The combination of silicone with methyl groups tends to make it extremely resistant to water yet it keeps them flexible and moving free, ideal properties for a lubricant. Dimethicone is viscoelastic meaning that, at high temperature, acts like a viscous liquid and, at low temperature, acts like elastic solid, similar to rubber. Due to the hydrophobic surface, when dimethicone is applied on hairs, it makes hairs shiny and slippery. The cosmetic ingredient review expert panel considered dimethicone poorly absorbed in to the skin due to the large molecular weight, so when applied on skin it prevents water loss and penetration of exogenous substances, thus acting as a skin protector. A bioengineering study evaluated the efficacy of 2% dimethicone as skin protecting lotion against sodium lauryl sulfate induced irritant contact dermatitis [20]. Dimethicone has been included in the list of substance commonly used to restore the impaired skin in humans [21, 22]. It is the first ingredient in foam formulated for relief of irritation from dermatosis, such as AD and allergic contact dermatitis. According to the food and drug administration (USFDA) dimethicone at concentrations between 1 and 30% is considered as a safe skin protectant. Dimethicone is well accepted in human medicine and a suitable alternative to the use of petrolatum which is greasy. Besides being water and UV resistant, dimethicone is not greasy and is not expensive. At this time it is not known if dimethicone is a beneficial treatment for dogs with AD.

Thus, the purpose of the present study was to investigate the efficacy of dimethicone as topical therapy in CAD. A solution composed by dimethicone (active ingredient) and cyclomethicone (vehicle) was made. Cyclomethicone is a clear, odorless, cosmetic solvent that is frequently used as vehicle or carrier of oil-soluble moisturizing products. It evaporates quickly and allows the active ingredient to start taking effect. For these reasons cyclomethicone was selected as vehicle for the preparation investigated in this study. Clinical signs were assessed using a validated scoring system (canine atopic dermatitis extent and severity index score (CADESI)) [23] and skin barrier was evaluated by measurement of TEWL. The hypothesis tested was that dimethicone would improve both skin barrier function and clinical signs in atopic dogs and that these two measurements would be correlated.

## 2. Material and Methods

All procedures used in this study were approved by the Institutional Animal Care and Use Committee of the University of Florida and a client consent form was signed prior to inclusion in the study.

### 2.1. Study Design

This study was a prospective, randomized, double-blinded, and vehicle-controlled study. The randomization was done by an assignment of numbers to each dog and blind hat draw.

### 2.2. Study Subjects

Eighteen privately owned dogs with AD were enrolled. All dogs were judged healthy on physical examination aside from skin disease and were clear of any secondary skin infections prior to enrolment. Diagnosis of AD was based on suggestive history, compatible clinical signs according to Prelaud criteria [24], and exclusion of other pruritic skin diseases that may mimic AD. 

The following inclusion criteria were adopted.

- Patients with a history of 1–6-year duration and mild to moderate severity AD based on a subjective clinical evaluation. The severity of AD was evaluated by the clinicians according to the following cut-off CADESI-03 values: remission: 0–15; mild AD: 16–59; moderate AD: 60–119; and severe AD: ≥120 as recommended by the International Task Force on Canine Atopic Dermatitis [25].
- Dogs were on the same diet for the entire study and no dietary changes were allowed once the dogs had been included in the study.
- Dogs with food as flare factor for AD were not eliminated as long as that component of the diet was controlled.
- Dogs had to be on flea control program starting at least 1 month prior to the inclusion in the study, to minimize the potential for clinical improvements due to flea control. This program included application of a flea repellant (e.g., ≥2% permethrin) at least monthly using special precaution in households where cats are present.
- Dogs were off all oral antihistamines, cyclosporine for a minimum of 1 month, essential fatty acid supplements, and long-acting injectable glucocorticoids for a minimum of 2 months while oral [26] and topical glucocorticoids for a variable time (5–14 days) [27]. The withdrawal time took into consideration also the type of glucocorticoids prescribed and the duration of treatment, as it would be done in clinical practice before intradermal skin testing. Medicated shampoos were not allowed during the trial. All dogs were not on allergen-specific immunotherapy, unless treatment had been started at least 1 year prior to the study. The dose and frequency could not be changed once enrolled in the trial.

The following exclusion criteria were also adopted.

- Dogs with exceptional severity of AD that did not allow the discontinuation of previous treatments were excluded regardless of the CADESI scores.
- Outdoor dogs were excluded due to the potential for environmental factors (e.g., rain) to decrease the residual effect of topical therapy.

### 2.3. Intervention

Dogs were randomly divided into two equal-size groups. Group A was treated with a skin protectant spray (dimethicone 2% as active ingredient and cyclomethicone as carrier) and group B was treated with vehicle only (cyclomethicone spray). Six body areas were treated. They included the concave surface of the pinnae, axillae, and inguinal area. In both groups the spray was applied by the owners at a dose of 2 pumps (equivalent to 1 mL/pump) per site, every day, for a total of 28 days. Both investigators and owners were blinded regarding the allocation to groups.

### 2.4. Clinical Assessment

Dogs were evaluated three times during this study: at baseline (day 0, before any topical application), 2 weeks (day 14 of treatment), and at the end of therapy (day 28 of treatment). Investigator's assessment was based on CADESI scores in conjunction with cytology. A validated version of the CADESI was used for this study [23]. In this scoring system, the body is divided into regions, and each region is evaluated and scored for a variety of clinical signs. The severity of each sign is evaluated on a scale from 0 to 3 corresponding to absent (0), mild (1), moderate (2), and severe (3). The total CADESI is calculated by adding the scores for each clinical sign and each body region. In the present study the mean of the concave surface of pinnae (right and left), axillary (right and left), and inguinal areas was considered for the regional CADESI assessment. Adverse effects were also recorded.

### 2.5. Cytological Assessment

Cytology from lesional areas was undertaken at each visit based on clinician's discretion. Scotch tape (3M, St Paul, MN, USA) was applied on the lesional skin and stained with Diff Quik (Baxter Diagnostics, McGraw Park, IL, USA). The tape was placed on a glass slide with the sticky side down and evaluated for the presence of bacteria and yeasts. As the number of bacteria or yeasts required for infection is variable and highly debated among clinicians, the diagnosis of relapsing secondary infection was based on both cytology and clinical assessment by the same clinician, who consistently assessed all cases. The cytology results were always correlated with the clinical presentation. Dogs relapsing with infections were eliminated from the trial and treated appropriately.

### 2.6. Skin Barrier Assessment: Transepidermal Water Loss Measurements (TEWL)

TEWL was measured using a closed-chamber evaporimeter (VapoMeter, Delfin Technologies Ltd, Kuopio, Finland) in an ambient temperature of 20–26°C. Dogs were allowed 30 minutes to acclimatize to the examination room prior to TEWL measurements. The assessment was done three times: at baseline (day 0, before any topical application), 2 weeks (day 14 of treatment), and at the end of therapy (day 28 of treatment). All TEWL readings were done in triplicate and the mean of the reading was used for statistical analysis. Three unclipped skin sites were evaluated: concave surface of pinnae, axillary, and inguinal areas. These sites have been selected since they are not only commonly affected sites but also sites for which a significant difference in TEWL has been reported between normal and atopic individuals [16].

### 2.7. Statistics

Pre- and posttherapy CADESI and TEWL measurements were compared using a mixed model ANOVAs. Relationships among these measurements were evaluated using Pearson product-moment correlations. All analyses were performed using the statistical software package SAS System for Windows version 9.0 (SAS Institute, Cary, NC, USA). A *P* value less than 0.05 was considered significant.

## 3. Results

### 3.1. Animals

**Figure 1:**
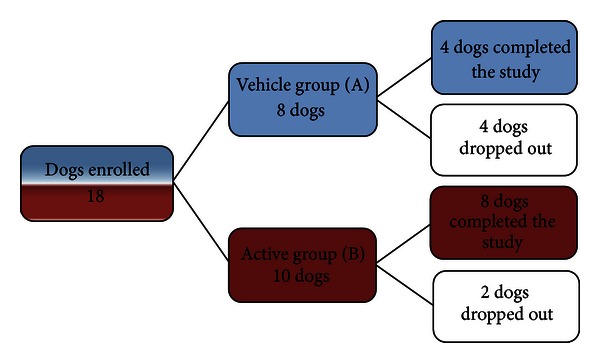
The flow chart shows the distribution of the dogs from the beginning to the end of the study.

Eighteen dogs were enrolled in the study and randomly allocated in the group A (vehicle group) and group B (active group). Ten dogs were in the active group and eight of them completed the study while eight dogs were enrolled in the vehicle group and only four of them finished the study. Six of eighteen dogs dropped out because five developed a superficial pyoderma and one presented *Malassezia* dermatitis on the left pinnae while enrolled in this study (Figure 1). The distribution of the dogs between the active and vehicle in two groups was homogeneous for age, age of onset disease, length of the coat, and sex (Table 1). Statistical analysis showed a lack of significant difference between the two groups at the beginning of the study (*t*-test; *P* > 0.05).

### 3.2. Clinical Evaluation

**Figure 2:**
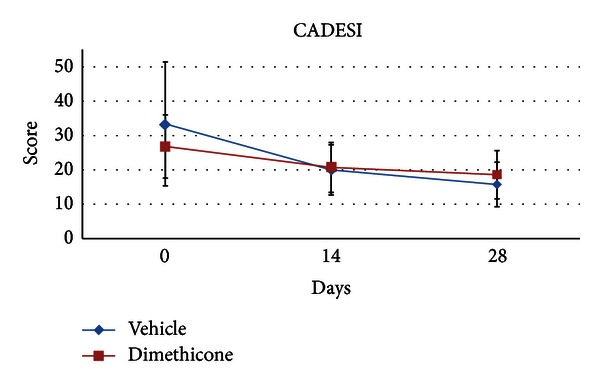
Mean total canine atopic dermatitis extent and severity Index (CADESI) scores as determined by the investigator. Error bars indicate ± standard deviation. ANOVA showed a significant effect of time with day 0 > day 28. The total CADESI was decreased for both the vehicle group and the dimethicone group, over time.

**Figure 3:**
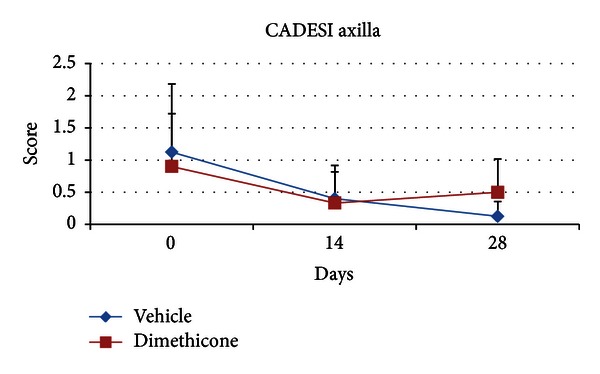
Mean regional Canine Atopic Dermatitis Extent and Severity Index (CADESI) of axilla. Error bars indicate ± standard deviation. ANOVA showed a significant effect of time, *P* < 0.005; day 0 > day 28. The axilla CADESI score was decreased for both the vehicle group and the dimethicone group, over time.

The total CADESI was decreased for both of groups over time of the study (ANOVA, *P* < 0.005; day 0 > day 28). CADESI score decreased in the active group from a mean of 26.8 (±9.2) to a mean of 18.6 (±7.03) and from 33.3 (±18.0) to 15.7 (±6.4) in the vehicle group (Figure 2). However, no effect of group or group × time interaction was found. For the regional CADESI, ANOVA showed only a main effect of region (axilla < groin < pinna) but no effect of group or group × time interaction was found (ANOVA, *P* < 0.005; day 0 > day 28). In particular, the axilla CADESI score in the active group decreased from 0.9 (±0.8) to 0.5 (±0.5) while in the vehicle group the same score dipped down from 1.1 (±1) to 0.1 (±0.2) (ANOVA, *P* < 0.005; day 0 > day 28) (Figure 3).

### 3.3. TEWL Measurement

**Figure 4:**
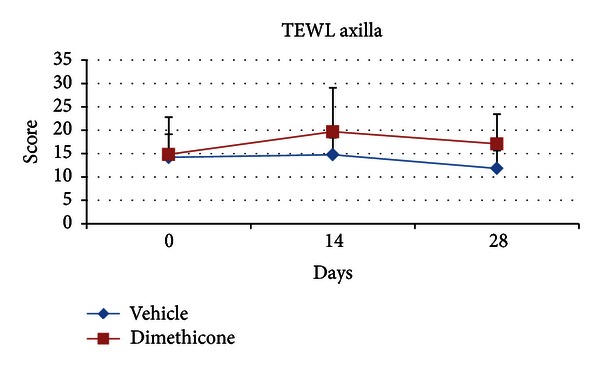
Mean regional transepidermal water loss (TEWL) axilla scores. Error bars indicate ± standard deviation. ANOVA showed a significant effect of time (*P* < 0.005), with day 0 > day 28. In the vehicle group the axilla TEWL score was slightly decreased while in the dimethicone group there was an increase over time of the study.

There was a decrease of the total TEWL from a mean of 12.4 g/m² hr (±2.1) to a mean of 11.1 g/m² hr (±2.0) in the vehicle group. This was in contrast to the active group in which the total TEWL increased from a mean of 12.5 g/m² hr (±2.5) to a mean of 15.7 g/m² hr (±2.4). However, there was no significant difference between the two groups and no effect of group or group × time interaction for the total TEWL (ANOVA, *P* < 0.005; day 0 > day 28). For the regional TEWL, ANOVA showed only a main effect of region (axilla > pinna > groin), but no effect of group or group × time interaction was found (ANOVA, *P* < 0.005; day 0 > day 28). In particular, the TEWL score from the axilla decreased from a mean of 14.2 g/m² hr (±4.9) to a mean of 11.8 g/m² hr (±3.8) in the vehicle group while in the active group the increase was from a mean of 14.8 g/m² hr (±7.9) to a mean of 17 g/m² hr (±6.3) (Figure 4).

### 3.4. Correlation between the CADESI and TEWL

No correlation was found between the total CADESI and the total TEWL (Pearson's *r* = 0.003; *P* = 0.7).

## 4. Discussion

In the present study daily application of topical dimethicone for 4 weeks in dogs with AD did not significantly improve clinical signs as assessed by CADESI and skin barrier as measured by TEWL. These results are different from what was reported in another study in humans [20] even though we used the same concentration of dimethicone and cyclomethicone. One possible explanation for the different results may be that the human study evaluated a protective effect of dimethicone in healthy volunteers after an acute insult rather than the ability to correct chronic changes, as the ones seen in patients with naturally occurring AD. The human study was also short lived (total duration of 5 days) rather than a longer study as the one that was done in our case.

The clear homogeneous solution used in our study was well accepted by owners, dogs, and clinicians. The cyclomethicone was chosen as vehicle, according to the clinical efficacy of a novel barrier protection cream, containing dimethicone and cyclomethicone in combination with aluminum-magnesium hydroxide stearate, for the treatment of eczema and hand dermatitis [28].

In the present study, the CADESI score decreased over time slightly for both groups but no effect for groups or group by time interaction was seen. At this time it is unknown if this result was due to a vehicle effect or due to a clinical benefit of both ingredients, dimethicone and cyclomethicone. A positive effect of vehicles has been reported in other studies such as in a clinical human study that compared a newly introduced barrier cream and its moisturizing vehicle, regarding skin compatibility [29]. Results showed no significant differences between the barrier cream and the vehicle. These results were explained by the improvement of the status of the skin and stratum corneum hydration seen in both groups. Consequently, the vehicle alone is capable of positively influencing skin hydration in some cases, but it was not specified if the improvement was linked to a visual cosmetic effect or to a true skin protection as detectable by TEWL. The lack of difference between the two groups observed in this study may indeed be due to the fact that cyclomethicone itself might have had some effect due to its emollient activity. Cyclomethicone is indeed silicone-based oil and it works in a variety of ways, a conditioning agent. The function of cyclomethicone in cosmetics is reported as antistatic, emollient, humectants, solvent, viscosity controlling, and hair conditioning [30]. When applied to skin and hair, cyclomethicone acts as a mild water repellent although the molecule is too big to penetrate the skin and therefore does not have a “true” moisturizing property. It is known to be able to evaporate quickly and not to be absorbed [31]. For this reason it is mostly used to carry and quickly deliver other ingredients. Nevertheless, it is perceived in human medicine to improve the softness and appearance of the skin and could have played a role in the improvement reported in the vehicle group. In our study there was a mild decrease of the total TEWL score in the vehicle group and no significant differences were found between the two groups and no effect of group or group × time interaction was detected. Measurement of TEWL in dogs has been reported to have limitations although it has been used successfully before to assess skin barrier function [16]. As in previous studies, a light restraint was used to minimize the movements [32] and the hair coat was manually parted just prior to placement of the probe to avoid the clipping [33]. The reproducibility of TEWL measurements was tested in a previously published study in which measurements were taken on the same dogs at different days and times [16]. In the present study, the same precautions were taken to minimize the factors that could interfere with the measurement of the TEWL such as allowing acclimation time, a controlled environment with consistent temperature and humidity, and a close chamber device which should be the least affected by ambient factors. Although the closed chamber devices are considered more reliable than the open chamber, limitations still exist and great variability can be found, and this makes it sometimes difficult to detect significant differences [34].

In the present study the results of the regional TEWL assessment were not correlated with regional CADESI scores. These results are in agreement with a compilation of studies in which no significant correlations were found between the TEWL assessment and the CADESI evaluation [35]. TEWL values have been reported to increase in proportion to the level of artificial damage of the skin [36]; however, for use in clinical studies, the significant site-to-site, day-to-day, and dog-to-dog variations make it very difficult for changes induced by disease, drugs, dietary supplements, or topical agents to be reliably detected as previously described by other investigators [34]. In order to reduce these variations, all dogs enrolled in our study were not affected by other disease except the mild AD, stayed on the same diet throughout the study, and were off drugs except flea control. Even if there are several limitations, TEWL remains the most helpful and noninvasive method used in many clinical trials to assess the skin barrier function in atopic dogs [37].

There are other limitations in this study. One of them is the group size. Based on the bioengineering human study and several pilot studies [20, 38, 39] regarding the topical treatment in atopic dogs, a group of eighteen dogs was chosen. It has revealed to be too small, limiting the power of the study. At the time of our study no pilot data was available; thus no calculation of power of analysis had been done prior to this study. In the future it would be interesting to test this product in a larger number of atopic dogs.

Another limiting factor was the high number of dogs dropping out of the study: 4 dogs of 8 (50%) dropped out in the vehicle group while only 2 dogs of 10 (20%) dropped out in the active group. Five dogs developed a superficial pyoderma and one dog *Malassezia* dermatitis on the right pinna during the treatment. These dogs were excluded from the study and treated appropriately. This study was performed from June to October; the hot and humid weather in association with the moisturizing treatment could have promoted the relapsing of pyoderma and *Malassezia* dermatitis in predisposed atopic dogs. Also, many of these dogs had a seasonal component to their allergies; thus they might have flared up with the infections as they were in the midst of their allergy season.

Another possible factor that affected the results was the duration and frequency of application of the product. This was a pilot study as no other studies had been done before regarding the application of silicon as skin protectants in atopic dogs. Thus there was no previous information regarding the frequency of application and the best protocol to use. In veterinary dermatology, topical therapeutic options are still somewhat limited in contrast to human medicine. According to previous studies [40, 41], significant ultrastructural and chemical changes occurred in the stratum corneum after just 3 weeks of topical treatment with an emulsion containing ceramides, free fatty acids, and cholesterol. In a study, clinical benefit was seen after 4 weeks of application [42]. Based on the bioengineering study, the efficacy of the dimethicone as skin protectant lotion was tested after 5 days [20]. As our study was a pilot study testing the dimethicone as skin protectant in atopic dogs, no specific data was available about the optimal treatment time. It would be useful to extend the treatment period in future studies.

Based on human studies topical moisturizer applications are best used as adjunctive therapy. In people with AD, it has been shown that routine use of a topical emollient can delay the need for topical glucocorticoid therapy [43]. Evidence suggests that the barrier creams are successful not only for the prevention of contact and irritant dermatitis [20, 42] but also in delaying the relapse of AD [44]. Thus, in hindsight, it might have been better to design a study to test the ability of dimethicone to help as an adjunctive therapy to increase time to relapse in animals that were in remission rather than to test its ability to control clinical signs without any additional medication. As skin barrier repair is an area of growing interest, it is important to consider the investigation of treatment options that can be useful for dogs with AD. Although the results of this pilot study did not show evidence of clinical benefit for the topical use of dimethicone, it is important to encourage alternative and innovative therapy to treat AD. Larger studies including more patients and for a longer period of time are desirable to better assess the long-term impact of skin barrier repair treatments and their benefit as adjunctive therapies for canine AD.

## Figures and Tables

**Table 1:** Allocation of the dogs between the vehicle group and dimethicone group according to age, onset of disease, length of the coat, and sex. Based on these parameters, the distribution was homogeneous between the two groups.

Parameter	Vehicle group (*n* = 8)	Dimethicone group (*n* = 10)
Age at study onset		
1–3 years	2	4
4–6 years	2	1
7–10 years	4	5
Onset of disease		
**<**2 years	1	3
**>**2 years	7	7
Length of the coat		
Short coat	8	7
Long coat	0	3
Sex		
Male	3	4
Female	5	6
